# Supplementary material for: Dose-response relationship between weekly physical activity level and the frequency of colds in Chinese middle-aged and elderly individuals
Source: PeerJ. 2024 May 29;12:e17459. doi: 10.7717/peerj.17459 (PMC11143968; doi:10.7717/peerj.17459)
Supplement: Supplemental Information 3 [file peerj-12-17459-s003.docx]

**Appendix A Questionnaire**

1. What is your gender? [Single choice question]

○ Male

○ Female

2. How old are you？ [Single choice question]

○Under 20 years old

○20-39

○40-59

○60-69

○Over 70 years old

3. Have you been diagnosed with any of the following chronic diseases at a level 2 or higher hospital [multiple choice]

□High blood pressure

□High Blood Lipid

□Diabetes

□ Malignant tumours such as cancer (excluding mild skin cancer)

□ Chronic lung diseases such as chronic bronchitis or emphysema (excluding tumours or cancer)

□ Liver disease (except fatty liver, tumour or cancer)

□ Cerebral stroke (stroke)

□ Kidney disease (excluding tumours or cancer)

□Stomach or digestive system diseases (excluding tumours or cancer)

□ Memory-related diseases (Alzheimer's disease, brain atrophy, Parkinson's disease)

□ Osteoarthritis or rheumatism or hospital-diagnosed osteoporosis

□None of the above diseases

4. Your level of education [multiple choice]

○Below junior high school

○High school

○Secondary school

○College

○Bachelor's degree

○Master's Degree

○Doctorate

5. Your occupation [optional]

○Heads of state organs, party organisations, enterprises and institutions

○Professionals and technicians

○Officials and related personnel

○Commercial and service personnel

○Agricultural, forestry, animal husbandry, fishery, and water conservancy workers

○Operators of production and transport equipment and related personnel

○Students

○Other workers who cannot be easily classified

○Military personnel

○Retirees

6. How many times per week have you exercised in the last year [multiple choice]

○No exercise (Please skip to Question 10)

○One to two times

○Three to five times

○Almost every day

7. The form of exercise you often choose when exercising [For ranking questions, please put the numbers in brackets]

[ ] Brisk walking

[ ] Running

[ ] Swimming

[ ] Basketball

[ ] Badminton

[ ] Square Dance

[ ] Taijiquan

[ ] Tennis

[ ] Skiing

[ ] Volleyball

[ ] Football

[ ] Table tennis

[ ] Strength Training

[ ] Yoga

[ ] Aerobics

[ ] Cycling

[ ] Others

8. How long have you been adhering to your primary form of exercise [radio question]

○ Within two months

○Two to three months

○Three to four months

○Four to six months

○Six months to one year

○Other _________________

9. How long do you exercise each time [single choice]

○Half hour

○1 hour

○1½ hours

○2 hours or more

10. Number of colds you have had in the past year [multiple choice]

○Almost never

○Once

○Twice

○Three times

○More than four times

11. Where do you usually exercise [For ranking questions, please put the numbers in parentheses]

[ ] Park Square

[ ] Community Open Space

[ ] Gymnasium

[ ] Public streets

[ ] Exercise areas provided by the community for specific purposes

[ ]Your own yard or indoor other

12. Do you have a professional to guide you in your fitness process [multiple choice questions]

○Yes

○No

13. How do you feel overall during your exercise [Single choice]

0 = same as in quiet state, 10 = exhaustion, can't continue exercise

○ As quiet as it gets (0 points)

○Very, very relaxed (1 point)

○Easy (2 points)

○ Moderate (3 points)

○Somewhat difficult (4 points)

○Difficult (5 points)

○(6 points)

○Very difficult (7 marks)

○ (8 points)

○ (9 points)

○Reaching the limit (10 points)

14. How does your heart feel during exercise [single choice]

○ As quiet as it gets (0 points)

○Very, very relaxed (1 point)

○Easy (2 points)

○ Moderate (3 points)

○Somewhat difficult (4 points)

○Difficult (5 points)

○(6 points)

○Very difficult (7 marks)

○ (8 points)

○ (9 points)

○Reaching the limit (10 points)

15. How does your breathing feel during exercise [single choice]

○ As quiet as it gets (0 points)

○Very, very relaxed (1 point)

○Easy (2 points)

○ Moderate (3 points)

○Somewhat difficult (4 points)

○Difficult (5 points)

○(6 points)

○Very difficult (7 marks)

○ (8 points)

○ (9 points)

○Reaching the limit (10 points)

16. How do your muscles feel during your exercise [Single Choice]

○ As quiet as it gets (0 points)

○Very, very relaxed (1 point)

○Easy (2 points)

○ Moderate (3 points)

○Somewhat difficult (4 points)

○Difficult (5 points)

○(6 points)

○Very difficult (7 marks)

○ (8 points)

○ (9 points)

○Reaching the limit (10 points)

17. Where are you located? [Single-choice question]

○Hebei Province

○Shanxi Province

○Liaoning Province

○Jilin Province

○Heilongjiang Province

○Jiangsu Province

○Zhejiang Province

○Anhui Province

○Fujian Province

○Jiangxi Province

○Shandong Province

○Henan Province

○Hubei Province

○Hunan Province

○Guangdong Province

○Hainan Province

○Sichuan Province

○Guizhou Province

○Yunnan Province

○Shaanxi Province

○Gansu Province

○Qinghai Province

○Taiwan Province

○Inner Mongolia Autonomous Region

○Guangxi Zhuang Autonomous Region

○Tibet Autonomous Region

○Ningxia Hui Autonomous Region

○Xinjiang Uygur Autonomous Region

○Beijing

○Tianjin

○Shanghai

○Chongqing

○Hong Kong Special Administrative Region

○Macao Special Administrative Region

**附件 A 运动方式调查问卷**

1. 您的性别 [单选题] *

| ○男 |
| --- |
| ○女 |

2. 您的年龄 [单选题] *

| ○20岁以下 |
| --- |
| ○20-39 |
| ○40-59 |
| ○60-69 |
| ○70岁以上 |

3. 您是否在二级以上医院被诊断有以下这些慢性病 [多选题] *

| □高血压 |
| --- |
| □高血脂 |
| □糖尿病 |
| □癌症等恶性肿瘤（不包过轻度皮肤癌） |
| □慢性肺部疾患如慢性支气管炎或肺气肿（不包括肿瘤或癌） |
| □肝脏疾病（除脂肪肝，肿瘤或癌外） |
| □脑卒风（中风） |
| □肾脏疾病（不包括肿瘤或癌） |
| □胃部疾病或消化系统疾病（不包括肿瘤或癌） |
| □与记忆相关的疾病（阿尔兹海默症，脑萎缩，帕金森症） |
| □骨关节炎或风湿或医院诊断的骨质疏松症 |
| □无上述疾病 |

4. 您受教育程度 [单选题] *

| ○初中以下 |
| --- |
| ○高中 |
| ○中专 |
| ○大专 |
| ○本科 |
| ○硕士 |
| ○博士 |

5. 您的职业 [单选题] *

| ○国家机关，党群组织，企业，事业单位负责人 |
| --- |
| ○专业技术人员 |
| ○办事人员和有关人员 |
| ○商业，服务业人员 |
| ○农，林，牧，渔，水利业生产人员 |
| ○生产，运输设备操作人员及有关人员 |
| ○学生 |
| ○不便分类的其他从业人员 |
| ○军人 |
| ○退休人员 |

6. 近一年来您每周运动锻炼次数是多少 [单选题] *

| ○不运动 (请跳至第10题) |
| --- |
| ○一到两次 |
| ○三到五次 |
| ○几乎每天 |

7. 您健身时经常选择的运动方式 [排序题，请在中括号内依次填入数字] *

| [ ]健步走 |
| --- |
| [ ]跑步 |
| [ ]游泳 |
| [ ]篮球 |
| [ ]羽毛球 |
| [ ]广场舞 |
| [ ]太极拳 |
| [ ]网球 |
| [ ]滑雪 |
| [ ]排球 |
| [ ]足球 |
| [ ]乒乓球 |
| [ ]力量训练 |
| [ ]瑜伽 |
| [ ]健身操 |
| [ ]自行车 |
| [ ]其他 |

8. 您坚持您主要锻炼方式的运动多久了 [单选题] *

| ○两个月以内 |
| --- |
| ○两到三个月 |
| ○三到四个月 |
| ○四到六个月 |
| ○半年到一年 |
| ○其他 _________________ |

9. 您每次运动时间长短 [单选题] *

| ○半小时 |
| --- |
| ○1小时 |
| ○1个半小时 |
| ○2个小时或以上 |

10. 您在过去一年中的感冒次数 [单选题] *

| ○几乎没有 |
| --- |
| ○一次 |
| ○两次 |
| ○三次 |
| ○四次以上 |

11. 您一般在哪运动锻炼 [排序题，请在中括号内依次填入数字] *

| [ ]公园广场 |
| --- |
| [ ]社区空地 |
| [ ]健身房 |
| [ ]公共街道 |
| [ ]社区专门提供的运动场所 |
| [ ]自家庭院或室内其他 |

12. 你在健身过程中是否有专业人士对您进行指导 [单选题] *

| ○有 |
| --- |
| ○没有 |

13. 您在运动锻炼过程中总体感觉 [单选题] *

0=跟安静状态下一样，10=力竭，不能继续运动

| ○和安静一样（0分） |
| --- |
| ○非常非常轻松（1分） |
| ○轻松（2分） |
| ○适中（3分） |
| ○有点困难（4分） |
| ○困难（5分） |
| ○（6分） |
| ○非常困难（7分） |
| ○（8分） |
| ○（9分） |
| ○到达极限（10分） |

14. 您在运动锻炼过程中心脏感觉 [单选题] *

| ○和安静一样 0分 |
| --- |
| ○1分 |
| ○2分 |
| ○3分 |
| ○4分 |
| ○5分 |
| ○6分 |
| ○7分 |
| ○8分 |
| ○9分 |
| ○达到极限 10分 |

15. 您在运动锻炼过程中呼吸感觉 [单选题] *

| ○和安静一样 0分 |
| --- |
| ○1分 |
| ○2分 |
| ○3分 |
| ○4分 |
| ○5分 |
| ○6分 |
| ○7分 |
| ○8分 |
| ○9分 |
| ○达到极限 10分 |

16. 您在运动锻炼过程中肌肉感觉 [单选题] *

| ○和安静一样 0分 |
| --- |
| ○1分 |
| ○2分 |
| ○3分 |
| ○4分 |
| ○5分 |
| ○6分 |
| ○7分 |
| ○8分 |
| ○9分 |
| ○达到极限 10分 |

17. 您所在的城市 [单选题] *

| ○河北省 |
| --- |
| ○山西省 |
| ○辽宁省 |
| ○吉林省 |
| ○黑龙江省 |
| ○江苏省 |
| ○浙江省 |
| ○安徽省 |
| ○福建省 |
| ○江西省 |
| ○山东省 |
| ○河南省 |
| ○湖北省 |
| ○湖南省 |
| ○广东省 |
| ○海南省 |
| ○四川省 |
| ○贵州省 |
| ○云南省 |
| ○陕西省 |
| ○甘肃省 |
| ○青海省 |
| ○台湾省 |
| ○内蒙古自治区 |
| ○广西壮族自治区 |
| ○西藏自治区 |
| ○宁夏回族自治区 |
| ○新疆维吾尔自治区 |
| ○北京市 |
| ○天津市 |
| ○上海市 |
| ○重庆市 |
| ○香港特别行政区 |
| ○澳门特别行政区 |
